# Masculinity and femininity in the divergence of male body image concerns

**DOI:** 10.1186/2050-2974-1-11

**Published:** 2013-03-28

**Authors:** Stuart B Murray, Elizabeth Rieger, Lisa Karlov, Stephen W Touyz

**Affiliations:** 1 The Redleaf Practice, 5 Redleaf Ave, Wahroonga, Sydney, NSW 2006, Australia; 2 Research School of Psychology, Australian National University, Canberra, ACT, 0200, Australia; 3 School of Psychology, University of Sydney, Sydney, NSW, 2006, Australia

**Keywords:** Male eating disorders, Gender role endorsement, Muscle dysmorphia, Anorexia nervosa

## Abstract

**Background:**

Given recent assertions suggesting that gender role endorsement may be relevant in the divergence of male body image concerns, this study examined the self-reported gender role endorsement in opposing dimensional extremes of male body image disorders, namely, muscle dysmorphia and anorexia nervosa. This study further examined the relationship between gender role endorsement and eating disordered and muscle dysmorphia disorder pathology.

**Methodology:**

Participants were 21 male muscle dysmorphia patients, 24 male anorexia nervosa patients, and 30 male gym-using controls from Australia, the United Kingdom, and the United States. All participants completed multidimensional measures of masculinity and femininity, and measures of eating disorder and muscle dysmorphia symptomatology.

**Results:**

Patients with muscle dysmorphia reported significantly elevated adherence to masculine (but not feminine) norms relative to control gym-using men and men with anorexia nervosa, whereas patients with anorexia nervosa exhibited elevated feminine (but not masculine) gender role endorsement relative to control gym-using men and men with muscle dysmorphia.

**Conclusions:**

Masculine and feminine gender role endorsement appear to be associated with the divergence of body image concerns towards muscularity and thinness-oriented ideals respectively.

## Background

With the increasing parity reported between males and females in both the prevalence and severity of body dissatisfaction [1], an increasing empirical focus has been directed towards illuminating the male experience of body image disturbance, given the dearth of empirical data relative to female body image disturbance [2]. Extant empirical data suggests that a large proportion of men in Western society experience body dissatisfaction [3,4] and pursue an ideal body that differs substantially from their current body [5]. Specifically, many men report a preference for a more muscular physique, which is consistent with the male body ideal frequently portrayed in contemporary Western media [4], although a smaller proportion of body dissatisfied men desire weight loss and reduced overall body mass [5,6].

This increasingly reported body dissatisfaction amongst males is said to facilitate the development of both anorexia nervosa [7] and muscle dysmorphia [8], which are two opposing dimensional extremes of body image psychopathology amongst males characterised by a pathological drive for thinness and muscularity respectively. Despite this increasing prevalence of male body dissatisfaction, and a well-documented bi-directional crossover between thinness- and muscularity-oriented body image disorders in men [4,9], little research to date has attempted to delineate the factors implicated in shaping the divergence amongst body dissatisfied males towards either thinness- or muscularity-oriented body image psychopathology.

Initial research has postulated that gay men may be more susceptible than heterosexual men to media images promoting thinness [10], experience a greater drive for thinness [11], and experience a greater risk for behavioural symptoms of thinness-oriented eating disorders [12]. However, contrasting findings have evidenced an elevated drive for both thinness and muscularity in gay males [13,14], whilst further research contends that gay males are no more likely to experience body dissatisfaction than their heterosexual counterparts [2,15]. Thus, sexual orientation may not entirely account for the directional divergence of body image psychopathology amongst body-dissatisfied males.

In contrast to sexual orientation, a recent review postulated that gender role endorsement may be helpful in delineating the factors implicated in the directional divergence of male body image psychopathology [16]. Gender role endorsement reportedly accounts for significantly greater variance in eating disorder symptomatology than sexual orientation [17,18], particularly in males [19]. As such, both heterosexual and gay men may be prone to thinness-oriented eating disordered symptomatology should they endorse lower masculine- and higher feminine gender roles [20], and males who endorse more feminine gender roles are also more likely to report an elevated drive for thinness than males who endorse more masculine gender roles [18]. Together, this body of research suggests a link between feminine gender role endorsement and thinness-oriented eating disordered symptomatology. Indeed, the ‘femininity hypothesis’ posits that stereotypically feminine traits such as dependence and passivity give rise to a tendency to seek approval from others and lowered self esteem, leading those endorsing feminine gender roles to pursue dietary restriction and purging behaviours in pursuit of what they perceive to be the ideal body [17].

In contrast, the endorsement of masculine gender norms is said to be protective against thinness-oriented eating disorders [18], yet masculine gender role endorsement has been reported to be relevant in the development of muscle dysmorphia [4], which is inclusive of muscularity-oriented disordered eating [21]. Research has found that elevated masculine behaviours and attitudes are predictors of an elevated drive for muscularity in men [22-24]. Furthermore, one study found that following failure on non-physical tasks when pitted against females, males were more likely to feel less physically capable and less muscular, which resulted in a compensatory drive for muscularity [25].

Thus, masculine and feminine gender role endorsement may occupy opposing roles in the divergence of body image psychopathology amongst males. However, a recent meta-analysis reported inconsistent findings depending on whether masculine and feminine gender role endorsement were conceptualised as unidimensional or multidimensional constructs [26]. Current research advocates the use of multidimensional measures of masculine and feminine gender role endorsement, which may better index the complex and multi-faceted nature of gender role endorsement [16]. Little research to date has examined masculine and feminine gender role endorsement as multi-faceted constructs in male samples [27], and no research to date has examined these constructs in clinical samples of male eating disordered patients.

The current research aims to investigate the relationship between feminine and masculine gender role endorsement and male presentations of anorexia nervosa and muscle dysmorphia, indexing whether these constructs are possible factors associated with the divergence of such contrasting forms of male body image disturbance. It is hypothesized that males with anorexia nervosa will endorse higher levels of feminine gender role endorsement than both control gym-using men and men with muscle dysmorphia, and that men with muscle dysmorphia will endorse elevated levels of masculine gender role endorsement than both control gym-using men and men with anorexia nervosa. In addition, it is further hypothesized that higher levels of feminine gender role endorsement will be associated with higher levels of eating disorder psychopathology, while higher levels of masculine gender role endorsement will be associated with higher levels of muscle dysmorphia psychopathology.

## Method

### Participants and procedure

The method of participant recruitment has been previously described in a larger study from which the present data set were derived (Murray et al., 2012). Male anorexia nervosa patients (*n* = 24) were recruited internationally from specialist eating disorder facilities in Australia (*n* = 14), the USA (*n* = 4), and the UK (*n* = 7), Male muscle dysmorphia patients (*n* = 21) were recruited from specialist treatment clinics (*n* = 14) and gymnasiums (*n* = 10) in Australia (*n* = 10), the USA (*n* = 8), and the UK (*n* = 6). Male gym-using controls (*n* = 30) were recruited from gymnasiums in Australia (*n* = 17), the USA (*n* = 3), and the UK (*n* = 10). All clinical participants were assessed and screened by clinicians experienced in eating disorders via semi-structured clinical interview to confirm current diagnoses and to exclude a prior history of anorexia nervosa (in the muscle dysmorphia condition) and muscle dysmorphia (in the anorexia nervosa condition), whilst gym-using control participants who responded to an advertisement at their gym were assessed and screened via a semi-structured clinical interview to exclude those with a history of either condition. Ethical approval was granted by the University of Sydney.

### Measures

*Conformity to Masculine Norms Inventory*[28]*(CMNI):* The CMNI is a 94-item self-report measure of endorsement of traditional masculine norms in dominant Western culture. Items are answered on a 4-point scale (0 = strongly disagree to 3 = strongly agree), with total CMNI scores ranging from 0 to 282. The CMNI comprises 11 subscales labelled Winning, Emotional Control, Risk-Taking, Violence, Dominance, Playboy, Self-Reliance, Primacy of Work, Power over Women, Disdain for Homosexuals, and Pursuit of Status. The CMNI shows good internal consistency (α = .94 for the total scale), three-week test-retest reliability (*r* = .95), and concurrent validity in terms of significant positive correlations with other masculinity-related measures [28]. In the present study the CMNI demonstrated good internal consistency (Cronbach’s *α* = .87).

*Conformity to Feminine Norms Inventory*[29]*(CFNI):* The CFNI is an 84-item self-report measure of endorsement of traditional feminine norms in dominant Western society. Items are answered on a 4-point scale (0 = strongly disagree to 3 = strongly agree), with total CFNI scores ranging from 0 to 252. The CFNI comprises eight subscales labelled Nice Relationships, Care for Children, Thinness, Sexual Fidelity, Modesty, Romantic Relationships, Domestic, and Investment in Appearance. The CFNI shows sound psychometric properties, yielding internal consistency of α = .88 for the total scale, showing a three-week test-retest reliability of .94, and showing a significant positive correlation with other well validated measures of femininity [29]. Of the subscales comprising the CFNI, the Thinness and Investment in Appearance subscales were removed from the analyses, due to being confounded with eating disorder symptomatology. In the present study the CFNI demonstrated good internal consistency (Cronbach’s *α* = .84).

*Muscle Dysmorphia Disorder Inventory*[30]*(MDDI):* The MDDI is a 13-item measure of muscle dysmorphia symptomatology, in which items are rated on a five-point Likert scale, with total MDDI scores ranging from 13–65. The MDDI measures three core components of muscle dysmorphia symptomatology: Drive for Size, Appearance Impairment, and Functional Impairment, which demonstrate good reliability (Cronbach’s α = .77-.85; test-retest reliability *r* = .81-.87), and construct validity [30]. In the present study the MDDI demonstrated good internal consistency (Cronbach’s *α* = .82).

*Eating Disorders Examination – Questionnaire*[31]*(EDE-Q):* The EDE-Q is a 36-item self-report questionnaire which measures the core behavioral and attitudinal features of eating disorders over the previous 28 days, and comprises diagnostic items as well as four subscales, namely, Dietary Restraint, Eating Concern, Weight Concern, and Shape Concern, with total scores ranging from 0 to 138. These subscales show good test-retest reliability [32] and good concurrent validity [33]. In the present study the EDE-Q demonstrated good internal consistency (Cronbach’s *α* = .85).

*Demographic Questionnaire:* This questionnaire obtained participant information pertaining to age, gender, sexual orientation, height, and weight.

### Statistical analyses

A series of planned contrasts were conducted to test for (i) differences between the clinical groups and (ii) differences between the control group and each clinical group, on the measures of conformity to masculine and feminine norms. In addition, correlational analyses were conducted to investigate the relationship between the measures of conformity to masculine and feminine norms and the measures of muscle dysmorphia and eating disorder symptomatology. Assumptions of normal variance of data were met, thus parametric testing was undertaken. The significance level was set at *p* < .05 for all tests.

## Results

### Participant characteristics

Among the 21 men with muscle dysmorphia, the mean age was 28.24 years (*SD* = 6.74; Range = 19–45); of the 24 men with anorexia nervosa, the mean age was 23.92 years (*SD* = 5.57; Range = 16–37); and in the control gym-using group, the mean age was 28.53 years (*SD* = 8.32; Range = 19–42). A one way ANOVA revealed that the mean ages within the three groups was not the same, *F*(2, 67) = 3.59, *p* = .033. An expected difference in body mass index (BMI = kg/m^2^) was also found (F(2,65) = 82.28, *p* < 0.001), such that those in the muscle dysmorphia group (mean = 32.40, *SD* = 4.75) demonstrated a significantly higher BMI than those in the anorexia nervosa group (mean= 17.45, *SD* = 2.10), *t(65)* = 13.47, *p* < .001, whilst the mean BMI of the muscle dysmorphia (mean = 32.40, *SD* = 4.75) and control groups (mean = 26.19, *SD* = 4.18) did not differ, *t(65)* = 4.72, *p* = .757*.* Chi squared analyses revealed no significant differences in sexual orientation between groups, *x*^*2*^(4, *n* = 70) = 4.82, *p* = .307.

### Conformity to feminine norms

Scores regarding the total scale and subscales of the endorsement of feminine norms measure (i.e., the CFNI) are shown in Table 1. The anorexia nervosa group reported significantly higher mean total scores on the CFNI than the muscle dysmorphia group, *t*(67) = 3.64, *p* = .001, and the control gym-using group, *t(*67) = 2.48, *p* = .016, although there was no significant difference between the muscle dysmorphia and control gym-using groups in this regard, *t*(67) = -1.29, *p* = .202.

**Table 1:** Correlation between the CMNI, CFNI, EDE-Q and MMDI

	**MDDI**	**EDEQ**
EDEQ	**.652**	-
CFNI	-.091	.136
Nice Relationships	-.101	.116
Involvement with Children	-.170	-.133
Sexual Fidelity	-.062	**.257**
Modesty	-.008	.212
Romantic Relationships	-.203	-.117
Domestic	.199	.200
CMNI	**.494**	.144
Winning	**.520**	.162
Emotional Control	**.406**	**.342**
Risk Taking	**.314**	.106
Violence	**.359**	.010
Power Over Women	**.462**	.095
Dominance	**.369**	**.238**
Playboy	.045	**-.240**
Self Reliance	**.396**	**.438**
Work	.105	.223
Disdain for Homosexuals	**.303**	.021
Pursuit of Status	**.280**	.133

Note: Bold text indicates *p *< 0.05.

Similarly, on the Sexual Fidelity and Modesty subscales of the CFNI, those in the anorexia nervosa group reported significantly higher scores than those in the muscle dysmorphia group, *t*(67) = 6.47, *p* = .000, *t*(67) = 5.70, *p* = .001, and the control gym-using group, *t*(67) = 5.25, *p* < .001, *t*(67) = 2.51, *p* = .014, although no differences were reported between those in the muscle dysmorphia group and the control group, *t*(67) = 1.46, *p* = .149, *t*(67) = .99, *p* = .327.

In terms of the Nice Relationships subscale of the CFNI, those in the anorexia nervosa reported significantly higher scores than those in the muscle dysmorphia group, *t*(67) = 3.06, *p* = .003, although there were no significant differences between control gym-using men and either the anorexia nervosa group, *t*(67) = 1.31, *p* = .195, or the muscle dysmorphia group respectively, *t*(67) = 1.83, *p* = .072. No significant differences were reported between the groups for the Romantic Relationship subscale, the Domestic subscale, or the Involvement with Children subscale of the CFNI.

### Conformity to masculine norms

Scores on the total scale and subscales of the conformity to masculine norms measure (i.e., the CMNI) are shown in Table 1. The muscle dysmorphia group reported significantly higher mean total scores on the CMNI than the anorexia nervosa group, *t*(67) = 4.73, *p* < .001, and the control gym-using group, *t*(67) = 4.94, *p* < .001, although there was no significant difference between the anorexia nervosa and control gym-using groups in this regard, *t*(67) = .18, *p* = .859. As shown in Table 2, this pattern was the same for the Winning, Risk Taking, Violence, Power over Women and Disdain for Homosexuality subscales.

**Table 2:** Mean (SD) scores on the CFNI and CMNI for the anorexia nervosa, muscle dysmorphia, and control gym-using groups

**Measures**	**Anorexia nervosa (N=24)**	**Muscle dysmorphia (N=21)**	**Gym users (N=30)**
	**Mean (SD)**	**Mean (SD)**	**Mean (SD)**
CFNI Total Score	122.42 (19.19) a	96.10 (34.03) b	105.07 (16.71) b
Nice Relationships	35.38 (8.81) a	28.10 (9.54) b	32.60 (5.22) a,b
Invest in Children	18.00 (7.97) a	16.48 (9.43) a	19.00 (5.63) a
Sexual Fidelity	21.86 (5.73) a	11.52 (6.38) b	13.67 (3.96) b
Modesty	17.04 (6.70) a	11.52 (5.48) b	12.93 (3.71) b
Romantic Relationship	15.10 (4.75) a	13.71 (4.61) a	14.40 (2.69) a
Domestic	15.29 (4.52) a	14.76 (5.03) a	14.13 (3.80) a
CMNI Total Score	111.71 (26.30) a	154.52 (34.80) b	111.80 (28.70) a
Winning	12.83 (5.26) a	20.10 (5.58) b	13.30 (4.30) a
Emotional Control	16.20 (6.99) a,b	17.05 (5.41) a	13.00 (4.57) b
Risk Taking	12.38 (5.82) a	17.00 (4.06) b	12.27 (5.97) a
Violence	9.54 (5.02) a	15.29 (4.73) b	10.47 (5.01) a
Power over Women	6.80 (3.87) a	13.10 (5.07) b	6.40 (3.62) a
Dominance	5.50 (2.55) a,b	6.48 (2.18) a	4.80 (1.74) b
Playboy	7.38 (4.69) a	16.81 (8.52) b	14.33 (4.37) b
Self Reliance	8.63 (3.87) a	8.76 (3.39) a	5.47 (2.80) b
Primacy of Work	10.63 (4.43) a	7.62 (3.20) b	8.60 (3.72) b
Disdain Homosexuals	14.00 (8.01) a	21.10 (7.34) b	13.87 (7.04) a
Pursuit of Status	9.17 (3.50) a,b	10.52 (2.88) a	9.06 (1.83) b

Note: Means with the same subscript do not differ at *p *= 0.05.

On the Emotional Control subscale and Dominance subscale of the CMNI, the muscle dysmorphia group reported significantly higher scores than the control gym-using group, although no differences were reported between the muscle dysmorphia and anorexia nervosa groups, nor between the anorexia nervosa and control groups. On the Playboy subscale of the CMNI, both the muscle dysmorphia, *t*(67) = 5.38, *p* < .001, and the control gym-using group, *t*(67) = 4.12, *p* < .001, reported significantly higher scores than the anorexia group, although there were no reported differences between the muscle dysmorphia and control gym-using groups, *t*(67) = 1.46, *p* = .150. In the Self Reliance subscale, both the muscle dysmorphia group, *t*(67) = 3.25, *p* = .002, and the anorexia nervosa group, *t*(67) = 3.23, *p* = .002, reported significantly higher scores than the control gym-using group, although there were no reported differences between the anorexia nervosa and muscle dysmorphia groups, *t*(67) = .13, *p* = .894.

On the Primacy of Work subscale, those in the anorexia nervosa group reported significantly higher scores than the muscle dysmorphia group, *t*(67) = 2.63, *p* = .011, and the control group, *t*(67) = 2.07, *p* = .042, but there was no difference between the control group and the muscle dysmorphia group, *t*(67) = .65, *p* = .516. No significant differences were reported between groups on the Pursuit of Status subscale.

### Correlational analyses

Pearson’s correlation coefficients were calculated to examine the association between muscle dysmorphia symptomatology and both masculinity and femininity and to examine the association between eating disorder symptomatology and both masculinity and femininity. As shown in Table 1, Muscle dysmorphia symptomatology was significantly positively correlated with most aspects of masculinity, but was not significantly correlated with any aspect of femininity. Eating disorder psychopathology was positively correlated with Sexual Fidelity, Emotional Control, Dominance and Self-Reliance, and was negatively correlated with Playboy.

## Discussion

The main aim of this study was to investigate the factors associated with diverging presentations of male body image concerns towards thinness- and muscularity-oriented extremes. In particular, we examined masculine and feminine gender role endorsement in men diagnosed with anorexia nervosa and men diagnosed with muscle dysmorphia, and a control group of gym-using men. As predicted, those with muscle dysmorphia generally reported significantly greater adherence to masculine gender roles relative to control gym-using men and those with anorexia nervosa, whereas those with anorexia nervosa reported significantly greater endorsement of feminine gender roles relative to those with muscle dysmorphia and to control gym-using men. These findings are consistent with the notion that masculine and feminine gender role endorsement may be associated with the divergence amongst body dissatisfied men towards pursuing more muscular or thinner body ideals [16,18].

In terms of feminine gender role endorsement, the men who reported the greatest global adherence to feminine gender norms were those with anorexia nervosa, whilst men with muscle dysmorphia did not differ from control gym-using men in global adherence to feminine norms. This finding is consistent with extant literature suggesting that feminine gender role endorsement may be a possible risk factor in the development of anorexia nervosa in males [18,34], although further illustrates that those pursuing hyper-muscular physiques do not endorse lesser feminine gender role adherence.

Furthermore, multiple facets of feminine gender role endorsement were elevated in men with anorexia nervosa (Sexual Fidelity, Nice Relationships, and Modesty), which is consistent with existing research pertaining to presentations of anorexia nervosa. For instance, the finding that sexual fidelity (e.g., “I would feel extremely ashamed if I had many sexual partners”) is elevated in anorexia nervosa appears consistent with research indicating low levels of sexual activity in individuals with anorexia nervosa [35] and may be part of the broader asceticism that has been documented in this population [36]. Furthermore, the finding that developing positive, supportive relationships as indexed by the Nice Relationships subscale (e.g., “Being nice to others is extremely important”) and the minimisation of the self’s needs within these relationships as indexed by the Modesty subscale (e.g., “I would feel uncomfortable being singled out for praise) are elevated in anorexia nervosa appears consistent with research documenting subjugation schemas [37], silencing the self, and conflict avoidance [38] in presentations of anorexia nervosa. However, several aspects of feminine gender role endorsement (Romantic Relationships, Involvement with Children, and Domestic) were not elevated in those with anorexia nervosa relative to those with muscle dysmorphia and control gym-using men. This unexpected finding likely requires further elucidation.

In terms of masculine gender role endorsement, global adherence to masculine norms was significantly elevated in those afflicted with muscle dysmorphia, whereas those with anorexia nervosa and the gym-using control men did not differ overall in their global adherence to masculine gender roles. This finding appears consistent with previous research illustrating that masculine gender role endorsement appears to be implicated in the pursuit of hyper-muscular physiques [4,39], although further suggests that the pathological pursuit of a thinner physique is not inclusive of lower levels of global masculine gender role adherence. Further supporting our hypotheses, the present findings illustrate that multiple facets of masculine gender role endorsement emphasising masculine power and force (i.e., Winning, Risk Taking, Violence, Power over Women, and Disdain for Homosexuals) are elevated in those with muscle dysmorphia relative to both those with anorexia nervosa and control gym users. With specific reference to disdain for homosexuals, perhaps an interesting endeavour for further research would be to explicate the relationship between this facet of masculine gender role endorsement and the drive for muscularity in both homosexual and heterosexual samples, in light of the presence of internalised homophobia in some facets of the gay male community.

However, several aspects of masculine gender role endorsement were comparable between muscle dysmorphia and anorexia nervosa, although significantly elevated relative to control gym using men (i.e., Emotional Control, Dominance, and Self Reliance). The elevated Emotional Control subscale scores in both clinical groups may reflect the similar affective regulatory features of dysfunctional behaviours such as excessive exercise and dietary restriction [21], whilst heightened levels of self reliance may emanate from an avoidance of perceived weakness/vulnerability in asking for help. For example, while yet to be examined in those with muscle dysmorphia, research has found that individuals with an eating disorder report lower levels of desired social support than healthy controls [40].

A surprising finding was that one aspect of masculine gender role endorsement (i.e., the Primacy of Work subscale) was actually higher in those with anorexia nervosa relative to those with muscle dysmorphia. This unexpected finding may relate to the perfectionistic tendencies and high achievement orientation consistently reported in those with anorexia nervosa [41].

The correlational data were strongly supportive of the hypothesized association between muscle dysmorphia and the endorsement of masculinity gender roles. Specifically, higher levels of muscle dysmorphia symptomatology (as indexed via the MDDI) were significantly correlated with total scores on the measure of masculine gender role endorsement masculine norm endorsement as well as most of the subscales of masculine norm endorsement. These significant correlations were generally of a medium effect size, with the correlation between the MDDI and the Winning subscale of a large effect size. Only three subscales of masculine gender role endorsement were not significantly correlated with muscle dysmorphia symptomatology, namely, the Playboy, Primacy of Work, and Pursuit of Status subscales (although the latter was marginally significant which may have reflected a lack of statistical power).

In contrast to muscle dysmorphia symptomatology, there were few significant correlations between either feminine or masculine gender role endorsement and eating disorder symptomatology as assessed by the EDE-Q. This may have been due to the fact that higher scores on the EDE-Q are typically reported by both men with anorexia nervosa and men with muscle dysmorphia [21], as both of these groups had significantly higher scores on the EDE-Q than the control gym-using men. Yet since these two groups showed different relationships with feminine and masculine norm endorsement, higher scores on the EDE-Q in turn did not demonstrate a clear relationship with CFNI and CMNI scores.

## Conclusion

Overall, the current findings therefore cast doubt on the notion that masculine gender role endorsement represents a protective factor against the development of eating disorders [18]. Rather, masculine gender role endorsement appears to be associated with the development of muscularity-related eating and body image concerns, although lesser masculine gender role endorsement may not necessarily predispose one to thinness-oriented concerns.

Although the present study is novel in demonstrating how various facets of masculine and feminine gender role endorsement are differentially related to the opposing dimensional extremes of male body image concerns, the study had several limitations. First, the small sample size, reflecting the rarity of these clinical presentations, limits the study’s power. Larger samples could also assess for possible differences in the association between male body image concerns and gender role endorsement across diverse ethnic groups since the current measure reflects Western concepts of masculinity and femininity. Second, the correlational design of the study precludes any causal inferences, and thus future research is needed to explicate the precise role of gender role adherence in the divergence towards opposing presentations of body image concerns amongst males. Further research may seek to replicate the present findings in larger community based samples of males, which may allow for more rigorous linear regression analyses of the predictive value of muscularity and femininity in shaping the directional divergence of body image concerns amongst males. Finally, the present study was limited by the fact that other forms of psychopathology (e.g., social anxiety and depression) were not screened out and thus may have contributed to the pattern of findings.

If replicated, the present findings could have significant treatment implications (i.e., the need to address beliefs regarding gender roles) for body image concerns amongst males. Given that treatment efficacy is enhanced through a thorough understanding of the psychopathological pathway into eating disorders [42], an increased understanding of additional factors implicated in the divergence of body image concerns amongst males is a noteworthy endeavour for future research. In this regard, the present findings suggest that the pathological pursuit of thinness appears to be generally inclusive of greater feminine gender role endorsement without reductions in masculine gender role endorsement, whereas the pathological pursuit of muscularity is generally inclusive of greater masculine gender role endorsement, without reductions in feminine gender role endorsement.

## Competing interests

The authors declare that they have no competing interests.

## Authors’ contributions

SM developed the study design, undertook data collection, and drafted the manuscript, ER supervised the design of the study and the drafting of the manuscript, LK undertook all statistical analyses, ST supervised the writing of the manuscript. All authors read and approved the final manuscript.
